# Na_3_V_2_(PO_4_)_3_ Cathode for Room-Temperature Solid-State Sodium-Ion Batteries: Advanced *In Situ* Synchrotron X-ray Studies to Understand Intermediate Phase Evolution

**DOI:** 10.1021/acs.chemmater.3c02585

**Published:** 2024-02-20

**Authors:** Bidhan Pandit, Morten Johansen, Cynthia Susana Martínez-Cisneros, Johanna M. Naranjo-Balseca, Belen Levenfeld, Dorthe Bomholdt Ravnsbæk, Alejandro Varez

**Affiliations:** † Department of Materials Science and Engineering and Chemical Engineering, Universidad Carlos III de Madrid, Avenida de la Universidad 30, 28911 Leganés, Madrid, Spain; ‡ Centre for Integrated Materials Research, Department of Chemistry, Aarhus University, Langelandsgade 140, DK-8000 Aarhus, Denmark

## Abstract

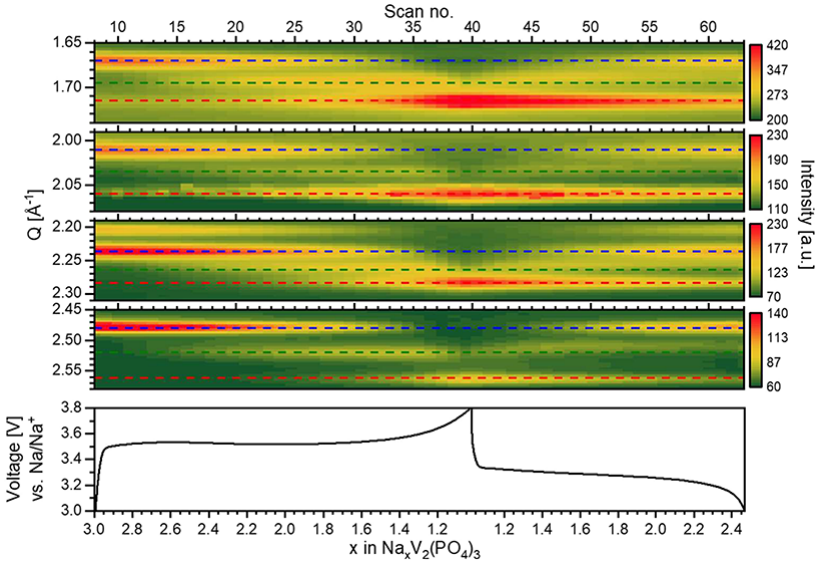

Sodium-ion batteries (NIBs) can use elements that are abundantly present in Earth’s crust and are technologically feasible for replacing lithium-ion batteries (LIBs). Hence, NIBs are essential components for sustainable energy storage applications. All-solid-state sodium batteries are among the most capable substitutes to LIBs because of their potential to have low price, great energy density, and consistent safety. Nevertheless, more advancements are needed to improve the electrochemical performance of the Na_3_V_2_(PO_4_)_3_ (NVP) cathode for NIBs, especially with regard to rate performance and operational lifespan. Herein, a core–shell NVP/C structure is accomplished by adopting a solid-state method. The initial reversible capacity of the NVP/C cathode is 106.6 mAh/g (current rate of C/10), which approaches the theoretical value (117.6 mAh/g). It also exhibits outstanding electrochemical characteristics with a reversible capacity of 85.3 mAh/g at 10C and a cyclic retention of roughly 94.2% after 1100 cycles. Using synchrotron-based *operando* X-ray diffraction, we present a complete examination of phase transitions during sodium extraction and intercalation in NVP/C. To improve safety and given its excellent ionic conductivity and broad electrochemical window, a Na superionic conductor (NASICON) solid electrolyte (Na_3.16_Zr_1.84_Y_0.16_Si_2_PO_12_) has been integrated to obtain an all-solid-state NVP/C||Na battery, which provides an exceptional reversible capacity (95 mAh/g at C/10) and long-term cycling stability (retention of 78.3% after 1100 cycles).

## 1 Introduction

Because of the recent surge in lithium’s price to an all-time high, there has been a significant push to replace lithium-ion batteries (LIBs) in many applications.^1^ As a result, manufacturers, policymakers, and researchers are paying sodium-ion batteries (SIBs) an unprecedented amount of attention.^2^ When assessing the benefits of building SIBs, the relative quantity of sodium in the Earth’s crust has always been important.^3,4^ These benefits have been expanded as SIB technology has advanced to include features including low-temperature performance, improved safety, reduced transportation difficulties, enhanced cell design, and more.^5,6^ As a result, SIBs have the potential for future technological advancements in the fields of electric cars (low-speed), grid-scale energy storage, and 5G station energy storage. Several nations are trying to establish governmental initiatives to promote SIBs to meet the decarbonization goals. Because of their mechanical strength insufficiency and combustible property, orthodox and commercial liquid organic electrolytes used in SIBs raise a safety concern. Nonflammable and mechanically and thermally stable solid electrolytes (SEs) may take the place of these liquid electrolytes.^7,8^

Organic polymer-based electrolytes and inorganic ceramic electrolytes are the two primary SE groups. The two main types of inorganic ceramic electrolytes are “Na superion conductor” (NASICON, Na_3_Zr_2_Si_2_PO_12_)^9,10^ and sulfide-based (Na_3_PS_4_)^11^ electrolytes. Among the abovementioned SEs, Na_3_Zr_2_Si_2_PO_12_ (NZSP) gets considerable attention because of its stability (normal, as well as high-temperature), higher ionic transfer number, broad electrochemical stability window, and mechanical strength.^12−14^ Hong and Goodenough et al. published groundbreaking studies in 1976^15,16^ on the solid solution Na_1+*x*_Zr_2_Si_*x*_P_3–*x*_O_12_ (NZSP, 0 ≤ *x* ≤ 3) with maximal conductivities of ∼10^–1^ S/cm at 300 °C for 1.8 < *x* < 2.2.^17,18^ The precise stoichiometry of NASICON composition exhibits the precise values of ionic conductivities, which may be challenging to manage when high-temperature sintering operations (approximately 1150 °C) are needed. It has since undergone substantial research as a solid electrolyte.

The adoption and utilization of SIBs will be remarkably influenced by the development of suitable cathode materials.^7^ Numerous innovative sodium-ion cathode materials have recently been prepared. Layered transition metal oxides and polyanionic compounds stand out among these cathode materials for their excellent electrochemical characteristics.^19,20^ Because of their propensity for irreversible structural phase changes and rapid water absorption, layered transition metal oxides must be kept in a glovebox. The stability, safety, and reversibility have all improved with polyanionic compounds.^21,22^ Because of its three-dimensional sodium mobility, excellent thermal stability, and quick ion conduction, Na_3_V_2_(PO_4_)_3_ (NVP) materials have drawn a lot attention among the polyanionic materials.^23^ NVP also offers a high redox voltage (3.4 V vs Na^+^/Na), an excellent theoretical energy density (almost 400 Wh/kg), and a little volume change as cathode for SIBs.^24^ However, NVP’s electrochemical performance is limited by its low intrinsic electrical conductivity.^25,26^

The low conductivity of NVP is often improved by surface treatment. To be more precise, combining these materials with carbon-based ones could result in a useful conductive framework that improves particle surface-to-surface electronic transit and allows pathways for ions and electrons to migrate.^27^ Because of the ideal properties of graphitized carbon, carbon is often considered as a great alternative to modify the electrical conductivity by providing conductive pathways to improve the electrochemical performance.^28^ With ascorbic acid as carbon source and reductant, the Na_3_V_2_(PO_4_)_3_/C electrode was effectively synthesized and it showed a high reversible capacity of 98 mAh/g at a current rate of 0.1 A/g and capacity retention of 74% after 450 cycles.^29^ Battery anode and cathode, both constructed by NVP/C composite, which was prepared by spray-drying and calcining, demonstrated an 80% capacity retention after 1543 cycles in 1 M NaClO_4_ in ethylene carbonate (EC)/dimethyl carbonate (DMC) (1:1) with 5 wt % fluoroethylene carbonate (FEC) electrolyte.^30^ Sodium-rich NVP cathode (Na_4_VP), which was prepared via a quick and easy chemical solution method, could supply additional sodium to compensate for the sodium loss. After 400 cycles of using NaPF_6_/diglyme electrolyte, the Na-free-anode full cell of the abovementioned electrode coupled with hard carbon showed an excellent capacity retention of 98.5%.^31^ In their review research, Zhang et al. examined the NVP cathode materials used in Na-ion batteries and discussed illustratively about the opportunities and problems facing NVP cathode advancement going forward.^32^

Using a solid-state method, we prepared hierarchical core–shell carbon-coated NVP/C particles with a highly conductive network between the surface and particles. Primarily, carbon coating on NVP particles may improve the electronic conductivity while reducing the diffusion paths of sodium ions. Amorphous carbon is also coated to the surface of the NVP, which not only improves the electrical conductivity but also prevents nanoparticle aggregation. To guarantee that all of the nanoparticles are electrochemically active, the carbon network that interconnects them also creates a fast electron transport network. Through high-rate capability (85.3 mAh/g at a current rate of 10C) and extremely stable cycle life (94.2% retention at a 2C rate), the NVP/C electrode demonstrates exceptional electrochemical features. Finally, utilizing the conventional mechanical milling followed by an annealing process, we have successfully synthesized a Na_3.16_Zr_1.84_Y_0.16_Si_2_PO_12_ electrolyte with low grain-boundary impedance, which resulted in total ion conductivity up to 0.202 mS/cm at room temperature. Furthermore, according to the electrochemical findings, Na_3.16_Zr_1.84_Y_0.16_Si_2_PO_12_ exhibits improved interfacial stability toward the Na cathode, and solid-state NVP/C||Na batteries with Na_3.16_Zr_1.84_Y_0.16_Si_2_PO_12_ SE exhibit longer cycle life up to 1100 cycles (at 2C). This study introduces novel, accurate, and flexible synthetic methods for SE preparation that are advantageous for real-world utilization in solid-state Na batteries.

## 2 Experimental Section

### 2.1 Synthesis of Na_3_V_2_(PO_4_)_3_/C

A SPEX ball mill (ball diameter 5 mm) was used to combine 10 mmol of V_2_O_5_ (Sigma-Aldrich, 98% purity), 30 mmol of NaH_2_PO_4_·2H_2_O (Sigma-Aldrich, 99% purity), and 2.5 g of sugar (C_12_H_22_O_11_, Merck, >99%) in a stainless steel vessel for 2 h at 1000 rpm.^33^ The mixture was then compacted into pellets (13 mm diameter, 1.5 mm thickness) by being calcined in argon atmosphere for 24 h at 800 °C.

### 2.2 Solid-State Electrolyte Preparation

The NASICON powder, with chemical composition Na_3.16_Zr_1.84_Y_0.16_Si_2_PO_12_, was prepared via standard solid-state procedure.^34,35^ Using a zirconia jar and balls (5 mm diameter), a stoichiometric amount of Na_2_CO_3_ (Sigma-Aldrich, 99.5% purity), (NH_4_)H_2_PO_4_ (Sigma-Aldrich, 98% purity), SiO_2_ (Sigma-Aldrich, 99.5% purity), and fully stabilized zirconia powder (8 mol % YSZ procured from Tosoh) were ball milled for 24 h at 350 rpm in ethanol. After the ethanol was removed (heated at 60 °C for overnight), the well-mixed product after ball milling was preheated in air for 4 h at 500 °C, then for 4 h at 800 °C, and lastly calcinated at 1100 °C (4 h).

To obtain NASICON pellets, a mixture consisting of calcined NASICON powder (49.6 wt %), ethanol/methyl ethyl ketone (MEK) (27.5 wt %, 50:50), benzyl butyl phthalate (BBP, 1.3 wt %), butyl phosphate (BP, 0.1 wt %, polyethylene glycol (PEG10000, 0.6 wt %), and polyvinyl butyral (PVB, 0.8 wt %) was ball milled at 400 rpm for 22 h at normal temperature in an agate jar containing agate balls (5 mm diameter). The resultant slurry was tape-casted over a piece of mylar foil and gradually dried for 24 h (in the air). The dried sheet was divided into tiny pieces, placed in a mold with the final pellet shapes, and uniaxially pressed for 30 min at 120 °C with an 80 kN force.^36^ The pellets were then sintered for 10 h in air at 1200 °C.

### 2.3 Electrode Fabrication, Coin Cell Assembling, and Electrochemical Characterization

A viscous slurry, including 70 wt % active material, 18 wt % conductive carbon (C65/vapor grown carbon fibers (VGCF) = 1:1), 12 wt % polyvinylidene fluoride binder, and *N*-methyl-2-pyrrolidone solvent was prepared to fabricate the working electrodes. The as-prepared slurry was homogeneously coated on clean aluminum foil after being thoroughly mixed in a planetary ball miller. The electrode (3–6 mg/cm^2^ active material) was followed by a 12 h vacuum drying period at 80 °C before being cooled to ambient temperature.

CR-2032 type coin cells were fabricated in an argon-filled glovebox (Jacomex) with a water/oxygen level less than 0.1 ppm. In order to serve as both the reference and counter electrodes, sodium metal thin disks were formed into a disk with a 12.7 mm diameter and attached to a stainless steel current collector. For comparison purposes and as reference, a half-cell based on a conventional liquid electrolyte 1.0 M NaClO_4_ in propylene carbonate (PC) solution infiltrated into a separator of glass microfibers (Whatman GF/D) was fabricated by applying 800 psi (5.5 MPa) of uniaxial pressure. For solid-state batteries, the proposed NASICON pellets were used as both electrolyte and separator in the same coin cell setup with a reduced uniaxial pressure of 600 psi.

Before performing electrochemical studies, the cells (associating with both liquid and solid electrolytes) were given a 12 h rest period at 25 °C to allow for full electrolyte penetration. At room temperature, tests of the rate performance and galvanostatic discharge/charge were performed across the voltage range of 3–3.8 V vs Na^+^/Na. The mass of the active electrode material is considered for determining all specific capabilities. All the details of material characterization are illustrated in Supporting Information S1.

### 2.4 *Operando* Synchrotron Powder X-ray Diffraction

The active material [Na_3_V_2_(PO_4_)_3_/C], carbon black (C65+VGCF), and binder polytetrafluoroethylene (PTFE) were mixed in a wt % ratio of 80:15:5 to construct cathode pellets for the *operando* synchrotron powder X-ray diffraction (SR-PXRD). The materials were mixed with acetone, which was subsequently evaporated. The obtained composite was pressed (at 1.8 tons) to a free-standing pellet 7 mm in diameter and a thickness of ∼200 μm. The free-standing cathode pellet was cycled against a Na metal anode using glass microfibers (Whatman GF/B) separator and 1 M NaClO_4_ in PC electrolyte. In an typical AMPIX battery test cell, the battery stack was mounted in order to inspect transmission X-ray scattering study.^37^

The AMPIX cell was linked to a BioLogic VMP3 potentiostat and installed on a specified diffractometer at the DanMAX beamline, MAX IV, Lund, Sweden, and galvanostatically cycled within 3 and 3.8 V at a current rate of 1C.

SR-PXRD data were collected using an X-ray wavelength of λ = 0.354130 Å and a DECTRIS PILATUS3 × 2 M CdTe area detector during battery charge–discharge. Powder patterns were collected with an exposure time of 3 s to acquire a pattern every 2 min. All PXRD data were azimuthal-integrated using the Data Analysis WorkbeNch^38,39^ using a calibration based on a LaB_6_ standard positioned at the cathode side in the AMPIX cell. The powder patterns were scaled to compensate for beam intensity fluctuations (Figure S2).

### 2.5 Sequential Rietveld Refinement of PXRD Data

The FullProf software^40,41^ working in sequential mode was used to refine the *operando* PXRD data using the Rietveld refinement technique. A linear interpolation among the manually chosen and refined points was used to define the background during refinement. Furthermore, a Thompson–Cox–Hastings pseudo-Voigt profile function was used to illustrate the Bragg peaks. Three structural models with varying sodium contents were used to describe the data: Na_3_V_2_(PO_4_)_3_ (space group: *R*-3*c*),^42^ Na_2_V_2_(PO_4_)_3_ (space group: *P*2_1_/*c*),^43^ and NaV_2_(PO_4_)_3_ (space group: *R*-3*c,* exchanging Ti for V).^44^ Each of the three phases were refined with respect to scale factor, unit cell parameters, and the profile parameters prior to sequential refinement where only the scale factors were refined for all phases while only refining unit cell parameters of Na_2_V_2_(PO_4_)_3_ phase. Because of the extensive peak overlap between the phases, it was not possible to refine unit cell parameters of Na_3_V_2_(PO_4_)_3_ or NaV_2_(PO_4_)_3_ simultaneously with Na_2_V_2_(PO_4_)_3_.

## 3 Results and Discussion

### 3.1 Structural and Morphology Characterizations of Na_3_V_2_(PO_4_)_3_/C

**Figure 1:**
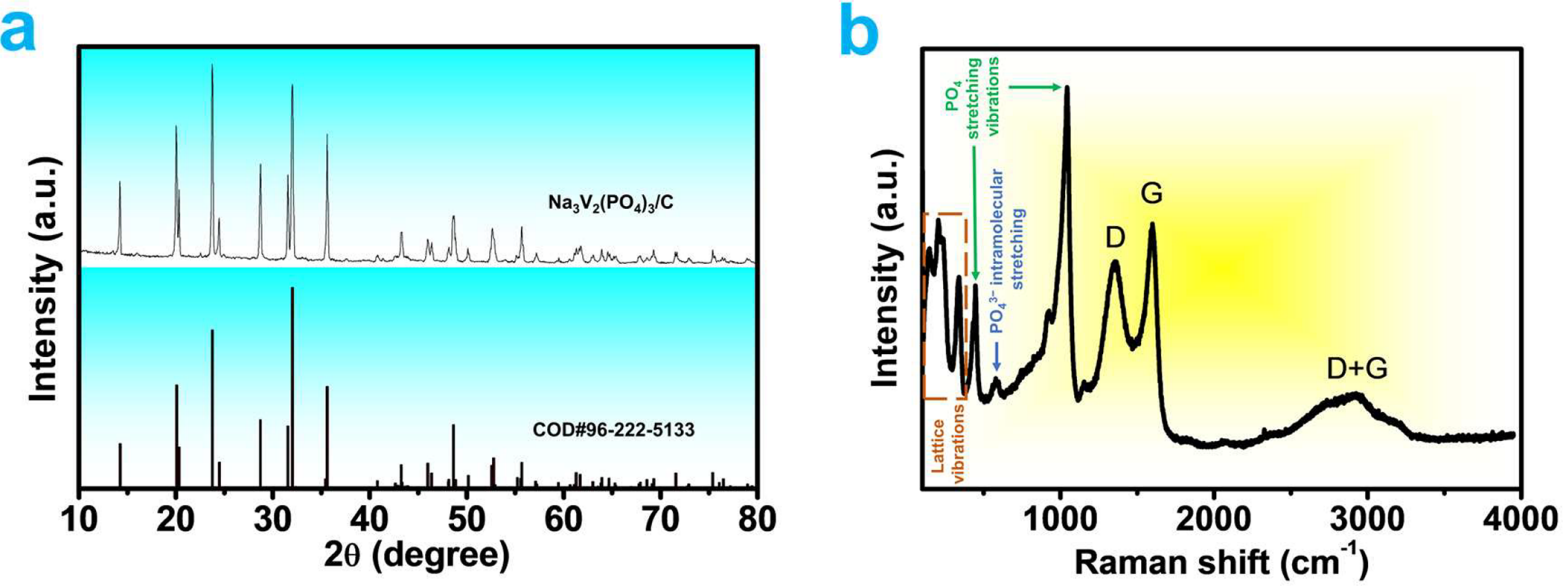
(a) PXRD pattern of Na_3_V_2_(PO_4_)_3_/C with a rhombohedral structure. (b) Raman spectrum of a Na_3_V_2_(PO_4_)_3_/C sample.

Figure 1a displays the PXRD pattern of as-synthesized NVP/C. The distinct and strong Bragg reflections indicate the rhombohedral Na_3_V_2_(PO_4_)_3_ structure (COD#96-222-5133, space group: *R*-3c) as the main phase. The characteristics of the carbon coating on NVP material was investigated using Raman Spectroscopy (Figure 1b). The crystalline lattice vibrations are thought to be the source of the prominent peaks at 138, 228, and 333 cm^–1^. Graphite’s E_2g_ vibrations (G-band) and disorder phonon mode (D-band), respectively, are each represented by a separate band at 1605 and 1350 cm^–1^.^45^ PO_4_ stretching vibration mode is similarly connected to the peaks at 446 and 1040 cm^–1^. The intramolecular stretching modes of the PO_4_^3–^ anion are responsible for the peak at 576 cm^–1^. The mixed phase, with a size between 2500 and 3500 cm^–1^, has a wide (D + G) bond, which includes both graphitic and amorphous carbon. Peak intensity ratios (*I*_D_/*I*_G_) of 0.85 for the D and G bands show how carbon is a little bit amorphous.

**Figure 2:**
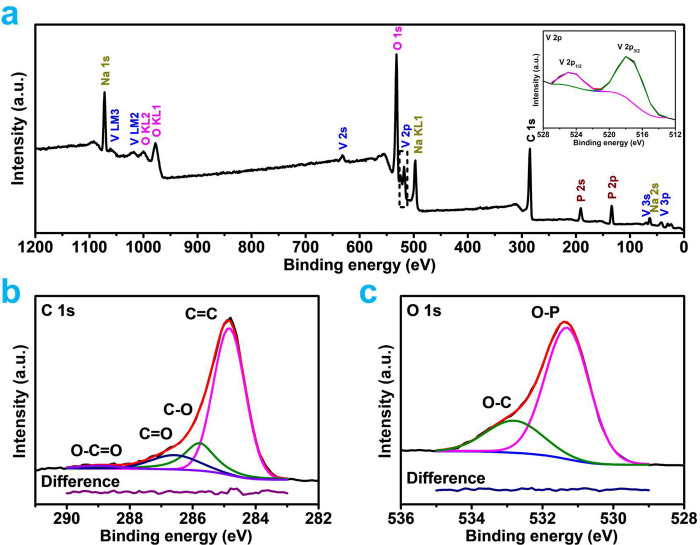
(a) XPS survey spectrum, inset shows V 2p core level spectrum, (b) C 1s, and (c) O 1s core level spectra of Na_3_V_2_(PO_4_)_3_/C.

**Figure 3:**
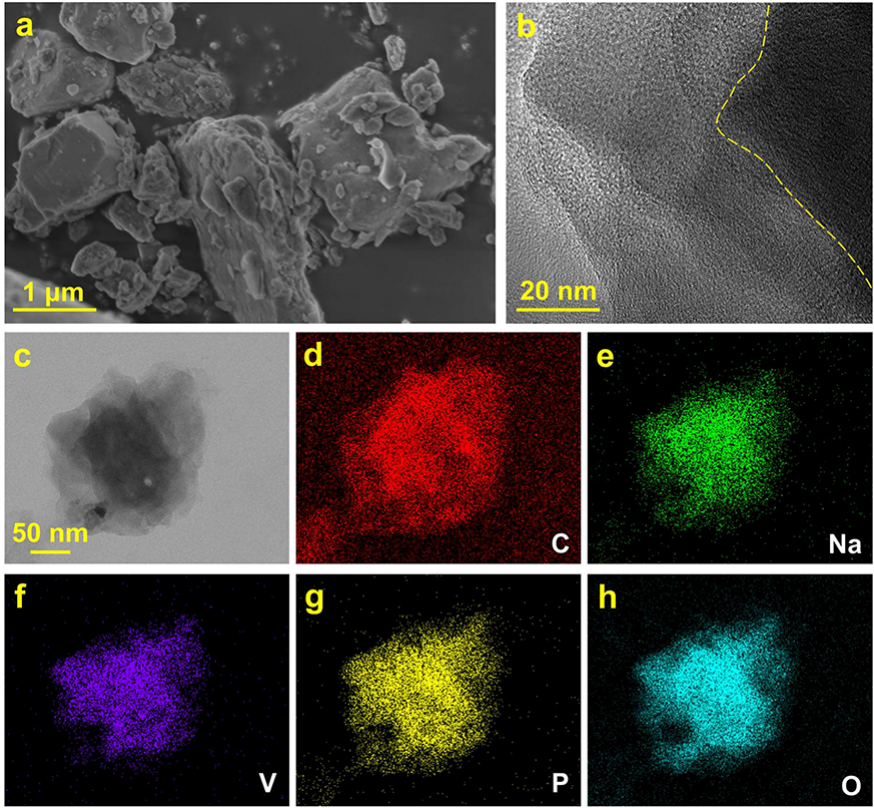
(a) FESEM image, (b) HRTEM image, and (c–h) EDS elemental mapping of NVP/C sample.

Data from X-ray photoelectron spectroscopy (XPS) was collected to examine the surface chemical compositions and bonding of NVP/C in more detail. The survey spectrum shown in Figure 2a confirms the existence of Na, V, P, and C elements, which is consistent with the findings of the elemental mapping in Figure 3c–h. The presence of V^3+^ species in the NVP/C sample is shown by the high-resolution V 2p spectra inset in Figure 2a, which exhibits two peaks at 523.9 and 516.9 eV that associate to V 2p_1/2_ and V 2p_3/2_, respectively.^46,47^ It should be highlighted that the absence of additional V 2p states proves the successful synthesis of pure NVP/C.

According to the C 1s spectra of NVP/C (Figure 2b), the sp^2^ graphite C=C bond is what causes the peak at binding energy of 284.8 eV. The carbon sp^3^, which has various C–O bonding arrangements, may be responsible for the other, higher energy peaks. The binding energies of C–O, C=O, and O=C–O bonds are confirmed by peaks 285.8, 286.6, and 289 eV, respectively.^48^ These results provide further proof that the outer layer of the NVP/C surface has been effectively coated with carbon, effectively increasing the electrical conductivity of the electrode material. Figure 2c shows the exact X-ray spectrum of the O 1s spectrum. The lattice oxygen of NVP may be responsible for the peak at 531.3 eV, while the peak at 532.8 eV may be caused by the O–C bond.^49^

Figure 3a depicts the prepared material’s morphology as assessed by field emission scanning electron microscopy (SEM) images. According to SEM, the size of NVP/C particles, which have a core–shell structure, ranges from 0.2 to 2 μm. The energy-dispersive X-ray spectroscopy (EDS) analysis and thermogravimetric data to determine amount of carbon loading are discussed in Figure S3 and Table T1. According to transmission electron microscopy (TEM) and high-resolution TEM (HRTEM), the as-synthesized material has a hierarchical structure that includes a carbon shell with a width of 40–100 nm, as revealed in Figure 3b. The carbon shell was formed on the NVP particle during the pyrolysis of sugar, which resulted in a clearly noticeable border between the shell and core. Furthermore, the TEM elemental mapping of NVP/C sample (Figure 3c–h) made it very evident how homogeneous the Na, V, P, and O elements were inside the particle core and C element outside the carbon shell.

### 3.2 Battery Performance of the NVP/C Electrodes

**Figure 4:**
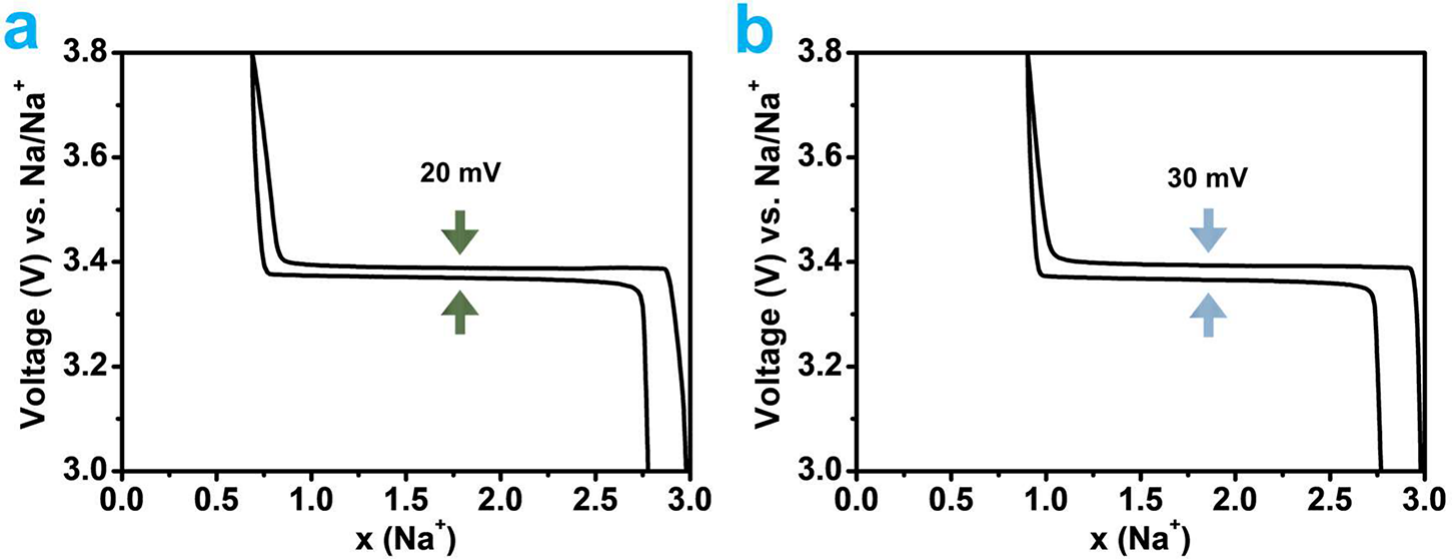
Electrochemical charge/discharge curves per Na^+^ ion in sodium half-cells at C/10 current rate for Na_3_V_2_(PO_4_)_3_/C electrode in half-cell vs Na in (a) 1 M NaClO_4_ in PC and (b) Na_3.16_Zr_1.84_Y_0.16_Si_2_PO_12_ solid electrolytes.

The coin-cell performance of the NVP/C cathode using both liquid (Figure 4a) and solid-state electrolytes (Figure 4b) was assessed at a C/10 rate (current density of 5.1 mA/g). In correspondence with typical biphasic reaction, the charge process at this specific current rate manifests as a long plateau at 3.39 V vs Na^+^/Na, which associates with deintercalation of almost 2 Na^+^, and the subsequent discharge process plateau around 3.37 V vs Na^+^/Na, which correlates with the reintercalation of approximately 2 Na^+^ (Figure 4a). At 3.4 V, the flat charge and discharge profiles of both cells correspond to the V^3+^/V^4+^ redox process in both batteries. The presence of the flat curves demonstrates the reversibility of the Na_3_V_2_(PO_4_)_3_ to NaV_2_(PO_4_)_3_ phase transition. At C/10 (1C = 117 mAh/g), the cell with the liquid electrolyte achieved a first cycle Coulombic efficiency of 90.4%, which is slightly higher than the solid-state battery’s 89% (Figure 4b). The battery assembled with the liquid electrolyte had the highest capacity and the lowest polarization (about 20 mV) by showing the smallest potential difference between two peaks. In the case of a solid-state battery, the polarization was somewhat greater at 30 mV. In both cells, the cycle starts with an irreversible capacity loss that cannot be overcome. This is due to the substantial electrolyte decomposition that results in the development of the solid electrolyte interphase (SEI).^50^

**Figure 5:**
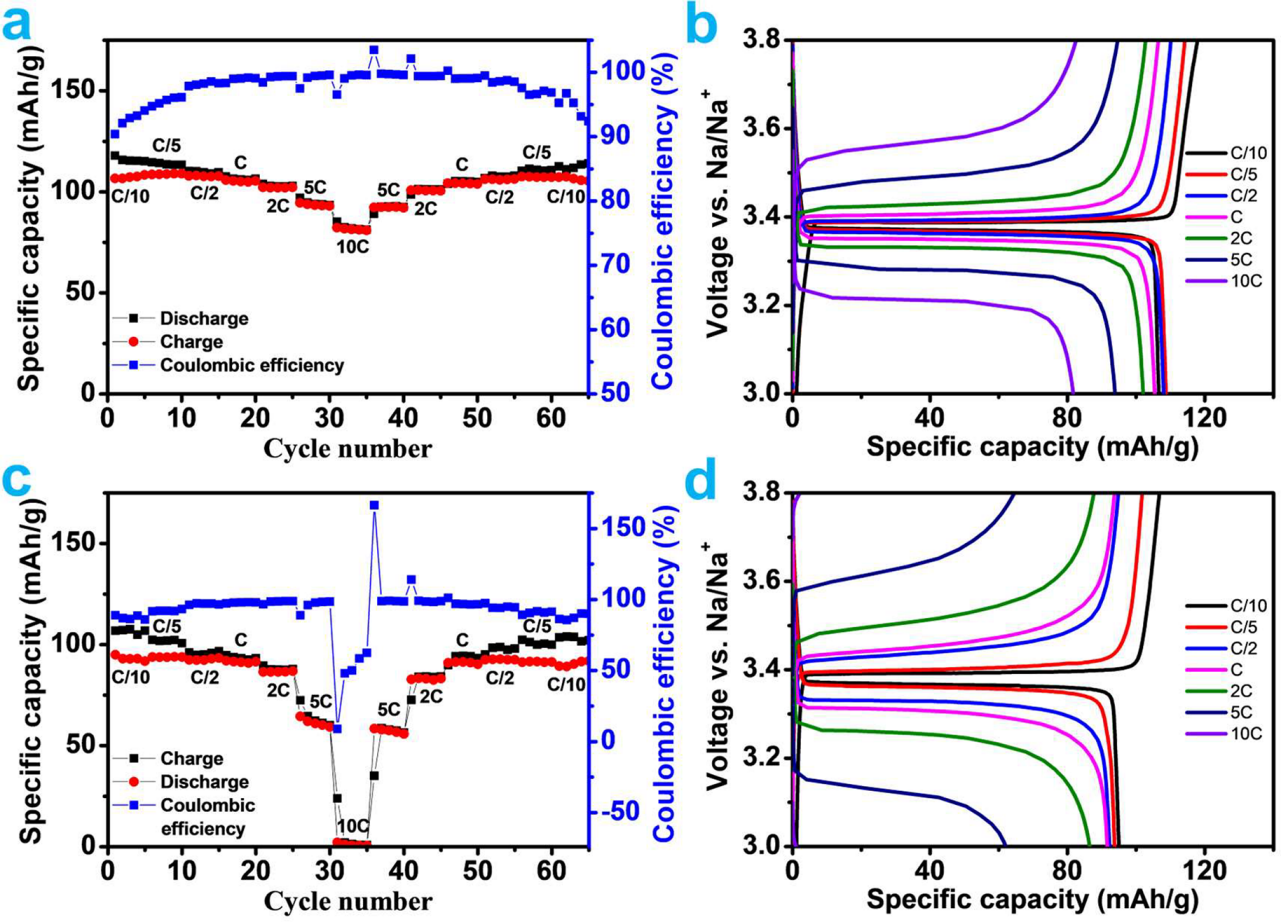
Rate performance (from C/10 to 10C) and galvanostatic charge/discharge profiles for Na_3_V_2_(PO_4_)_3_/C electrode in half-cell vs Na in (a,b) 1 M NaClO_4_ in PC and (c,d) Na_3.16_Zr_1.84_Y_0.16_Si_2_PO_12_ solid electrolytes.

Figure 5 shows the rate performance of both cells on the basis of the discharge and charge curves at various C rates. After considering the weight of the carbon in the composite, both NVP/C composites achieved specific discharge capacities of around 106.6 mAh/g at C/10, which is near the NVP theoretical capacity (117.6 mAh/g). A typical cell operating at 2C has a 4% decrease in specific discharge capacity, which results in 102.2 mAh/g. The association of the carbon coating to allow for further electrical integration really improved the capacity of NVP. After the 2C rate was mixed with solid-state electrolyte, the rate performance began to alter. It delivered 86.5 mAh/g at 2C and 64.5 mAh/g at 5C. The liquid electrolyte battery could still provide 82.3 mAh/g of capacity at 10C, which is excellent. According to Figure 5a,c, NVP/C may provide a much higher capacity using the liquid electrolyte design because it has better kinetic characteristics than a solid electrolytes cell, particularly at high current rates where high cation diffusion is required. However, the solid-state device’s capacity significantly dropped when the C rate was increased (to 10C, the highest rate used in the test). Figure 5b,d shows the capacity cycles at various C rates. All solid-state batteries presented a stable cycling at rates below 2C. There is a small rise of voltage polarization at the high current rates in both cases, and it is higher in the solid-state battery. At 5C and above, voltage loss is severe because of the increased polarization, and discharge and charge capacities start to vary quite a bit. However, after cycling at various current rates, all samples were able to recover their C/10 capacity, thereby demonstrating the robustness of the NVP/C structure.

**Figure 6:**
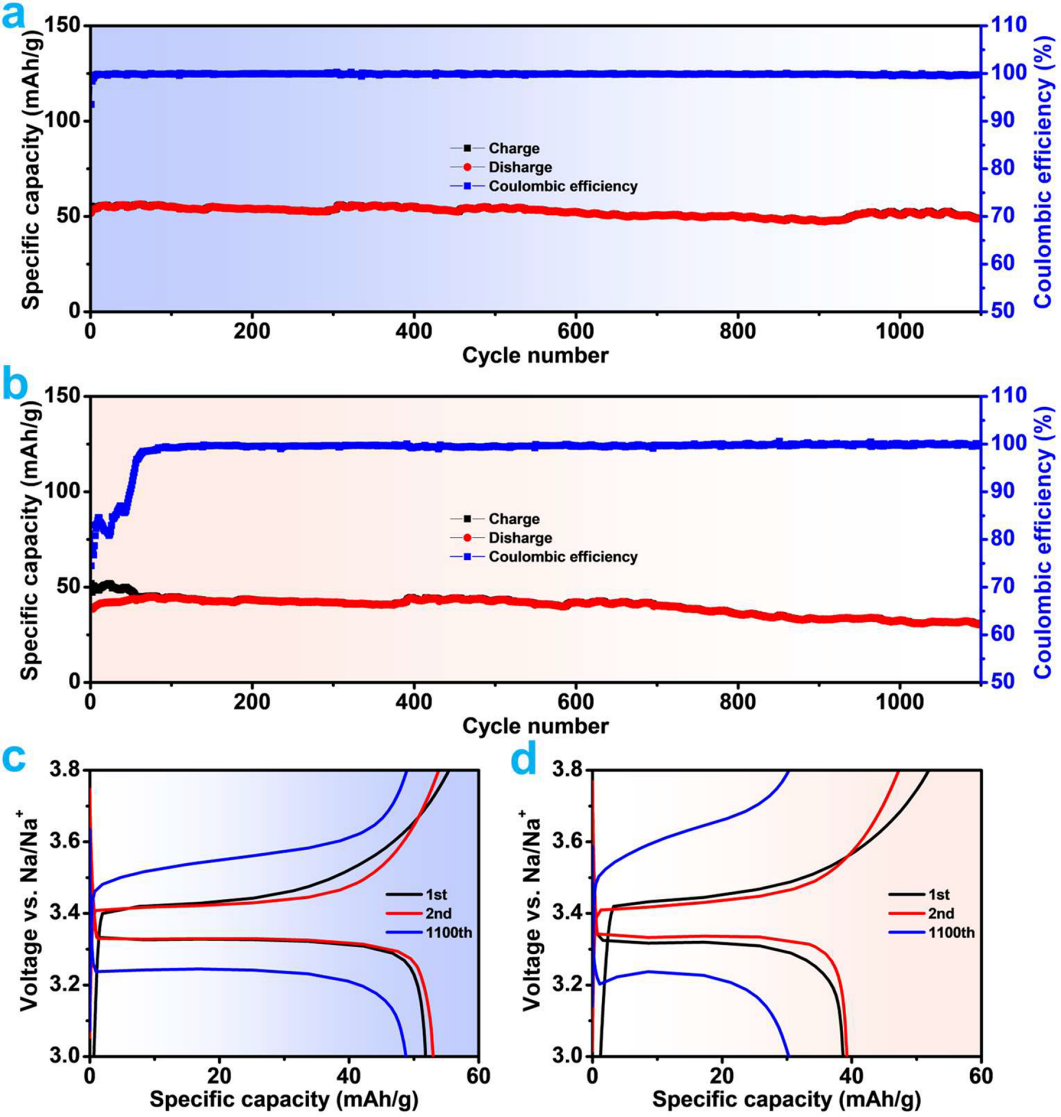
Stability studies and charge–discharge profiles at 2C rate for Na_3_V_2_(PO_4_)_3_/C electrode in half-cell vs Na in (a,c) 1 M NaClO_4_ in PC and (b,d) Na_3.16_Zr_1.84_Y_0.16_Si_2_PO_12_ solid electrolytes.

Figure 6 compares the long-term cycling performance of the NVP/C electrode in both electrolytes. For this test, the cells underwent 1100 continuous cycles at relatively fast current rate of 2C rate. The use of conductive carbon enhances cyclic stability in addition to increasing ionic mobility. For liquid- and solid-state batteries, the average Coulombic efficiencies at the 2C rate for 1100 cycles were 99.9% and 99.4%, respectively (Figure 6a,b). NVP/C electrode was able to retain 94.2% of its original discharge capacity while utilizing liquid electrolyte after 1100 cycles (Figure 6a) but only 78.3% when using solid-state electrolyte (Figure 6b). In comparison with the first cycle Coulombic efficiency in solid electrolytes (74.5%), the NVP/C electrode shows a higher 93.5% Coulombic efficiency in the liquid electrolyte battery. Additionally, the Coulombic efficiency immediately increases and remains constant during the whole battery cycling and achieves around 100% in the case of the liquid electrolyte cell. Conversely, the solid-state battery needs a few more cycles to reach 100% Coulombic efficiency (≈100 cycles). This is because the solid electrolyte–electrode interface is not well formed or fully optimized, which results in increased resistance, hindered ion transport, and reduced overall Coulombic efficiency of the battery. Figure 6c,d shows the discharge and charge curves from selected cycles of prolonged cycling at the 2C rate. The discharge and charge curves of NVP/C did not significantly alter with cycling in both batteries, which indicates the excellent stability of electrode. Although only a very little loss of capacity is seen, as shown in Figure 6c, the difference between the charge and discharge redox voltages of NVP/C hardly altered throughout the course of 1100 cycles and only rose from 100 mV (1st cycle) to 297 mV (1100th cycle). In a solid-state battery (Figure 6d), the polarization increased with cycling at a significantly faster rate (from 125 to 405 mV). This demonstrates the efficacy of our solid-state battery since it shows excellent results compared with earlier solid-state battery studies based on NVP/C electrodes (Table T2) and has a longer cycle performance even at room temperature. The excellent performance of the solid-state battery corresponds to the good ionic conductivity of the prepared SE.^51^

### 3.3 Structural Phase Transitions and Reaction Mechanism

The sodium battery’s cathode, NVP/C, follows the reactions shown below during charge and discharge, respectively:
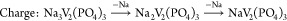
1
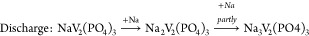
2

Rietveld refinement on the PXRD data was carried out using the NASICON-type Na_3_V_2_(PO_4_)_3_ structural model with the space group of *R*-3*c* (Figure S4). The NVP/C electrode shows lattice parameters for Na_3_V_2_(PO_4_)_3_ of *a* = 8.729(2) Å, *c* = 21.80(8) Å, and *V* = 1438.78(7) Å^3^, which are in agreement with the earlier reported articles.^52−56^

**Figure 7:**
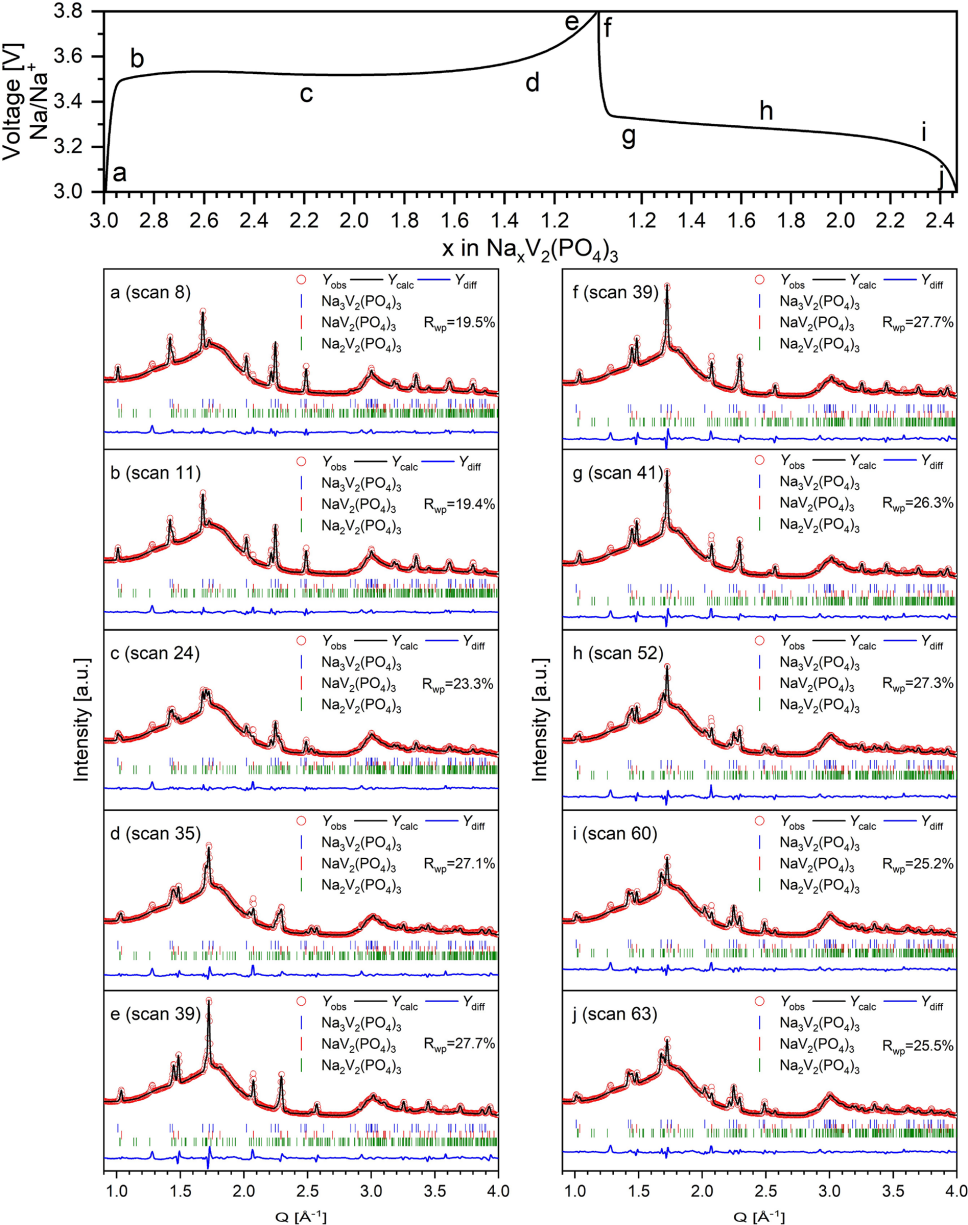
*Operando* PXRD spectra with 3-phase Rietveld refinement at specific charge–discharge points. Nonindexed reflections at 1.3 and 2.1 Å^–1^ are from additives used in the cathode pellets and the Na metal anode, respectively.

*Operando* PXRD spectra were obtained from the cathode during galvanostatic charge/discharge in 3–3.8 V voltage window at higher 1C rate. Hence, the NASICON structure is preserved throughout battery performance, as shown by the fact that the general patterns stay the same, and the selected peaks reversibly evolve during the electrochemical activities (Figure 7). The reversibility of the phase transitions during ongoing electrochemical activity is very helpful for the mechanical stability of the material. The structural reversibility is confirmed from the observations in the *operando* PXRD data.

**Figure 8:**
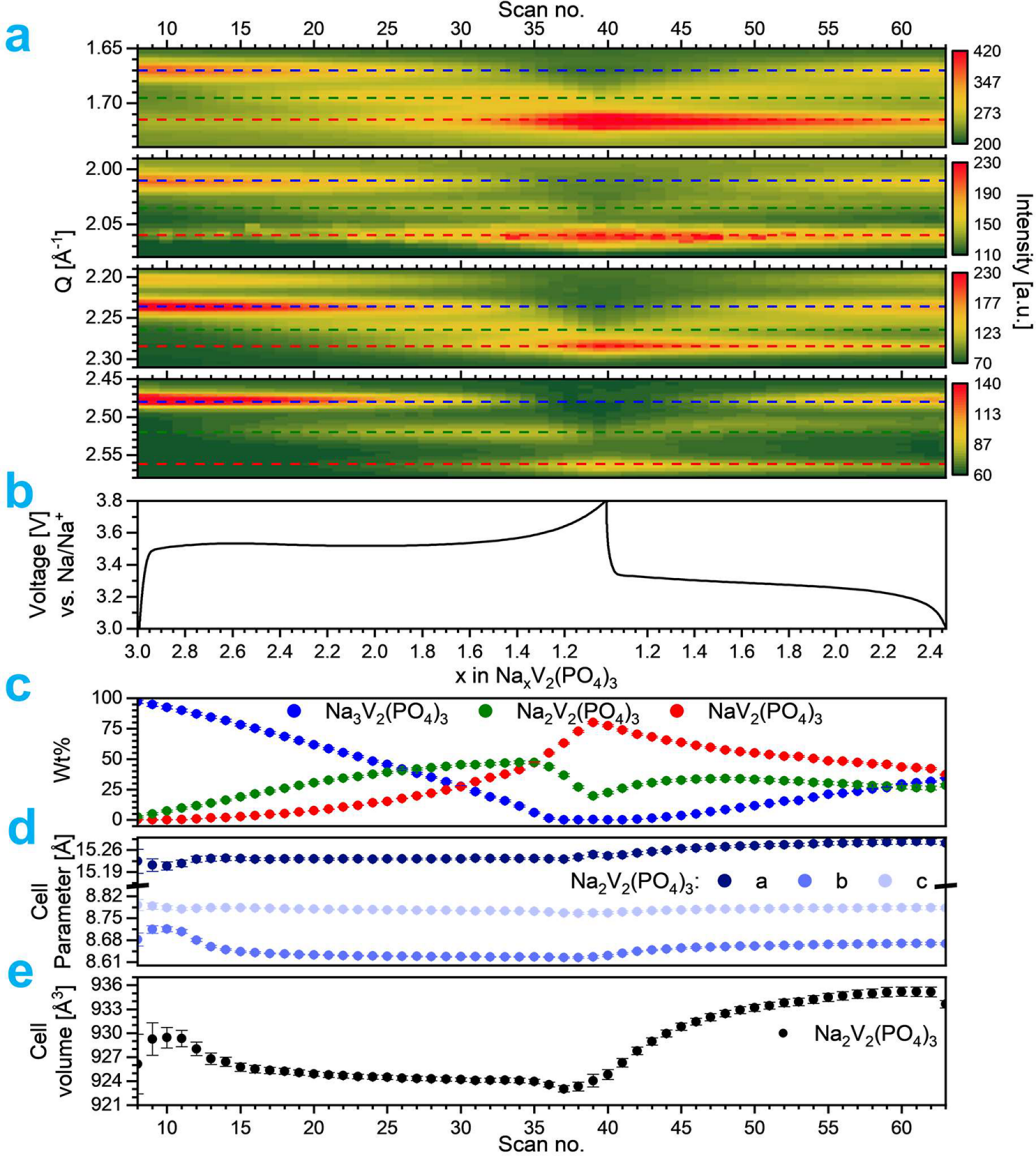
(a) Overview plot of acquired scattering data collected from *operando* synchrotron PXRD indexed using coloration of dotted lines for Na_3_V_2_(PO_4_)_3_ (blue), Na_2_V_2_(PO_4_)_3_ (green), and NaV_2_(PO_4_)_3_ (red). (b) Associated galvanostatic charge/discharge data. (c) Extracted wt % from Rietveld refinement. (d) Refined unit cell parameters for the Na_2_V_2_(PO_4_)_3_ phase. (e) Refined unit cell volume of Na_2_V_2_(PO_4_)_3_.

To analyze the associated volume variation and structural, as well as phase, transformation of Na_3_V_2_(PO_4_)_3_ cathode, *operando* PXRD (Figure 8a) spectra were analyzed, as well. In Figure 8, simultaneously recorded galvanostatic charge/discharge behavior (Figure 8b) is directly related to the structural changes observed from the *operando* PXRD data (Figure 8c–e).

The *operando* PXRD reveals an important first-order phase transition at the starting of sodium extraction during the charge process where the primary Na_3_V_2_(PO_4_)_3_ phase peaks slowly diminish, and new peaks slowly emerge at higher *Q* values corresponding to a smaller unit cell. As previously suggested from eqs 1 and 2, sodium extraction of Na_3_V_2_(PO_4_)_3_ occurs through two discrete, flat redox plateaus at around 3.37 and 3.39 V. The discharged state, NaV_2_(PO_4_)_3_, is obtained after extracting 1.5 Na ions per formula unit (f.u.) and is well described by a rhombohedral NASICON structure with a smaller unit cell. Before forming the desodiated phase, NaV_2_(PO_4_)_3_, an intermediate monoclinic distorted Na_2_V_2_(PO_4_)_3_ NASICON is formed around 0.4 h or after extracting ∼0.5 Na pr. f.u. Upon further desodiation, the NaV_2_(PO_4_)_3_ structure is slowly formed, and the Na_3_V_2_(PO_4_)_3_ structure slowly disappears while the amount of Na_2_V_2_(PO_4_)_3_ is unchanged. The three phases appear to coexist and can be used to model the system when the *x*-value in Na_*x*_V_2_(PO_4_)_3_ is between 1.2 and 2.5 (Figure 8b). When fully charged, Na_3_V_2_(PO_4_)_3_ and Na_2_V_2_(PO_4_)_3_ have almost been converted completely into the desodiated NaV_2_(PO_4_)_3_ phase. During Na intercalation (discharging), the intermediate Na_2_V_2_(PO_4_)_3_ phase is not as visible from the diffraction patterns as during the charge process. However, the solid solution behavior of the intermediate phase is once again observed with the unit cell gradually expanding until it rearranges into a fully sodiated structure. Upon Na intercalation, the unit cell expands beyond the size of the structure during charging, which could indicate that the Na ion intercalation mechanism is different from the extraction mechanism as seen by Sørensen et al. on Li_3_V_2_(PO_4_)_3_.^57^

The behavior of a Na solid solution would be indicated by the absence of an abrupt shift in the angular peak positions. It is in line with past electrochemical and computational investigations that the Na_3_V_2_(PO_4_)_3_ electrode experienced a two-step biphasic transition during fast cycling.^43,58^ When cycling at this rate, it is not uncommon to observe slightly delayed structural progress and that some materials start to exhibit some solid solution behavior, as is observed with the intermediate, Na_2_V_2_(PO_4_)_3_, phase.

The evolution in phase fractions (wt %) for Na_3_V_2_(PO_4_)_3_, Na_2_V_2_(PO_4_)_3_, and NaV_2_(PO_4_)_3_ are shown in Figure 8c. The refined unit cell parameters and unit cell volume from Figure 8d,e depict that the Na_3_V_2_(PO_4_)_3_ with a high Na content exhibits some solid solution reaction mechanism (constantly varying unit cell parameters), which suggests that a limited number of Na ions may be extracted before phase transition occurs during electrochemical cycling at high current rate. The refined parameters and unit cell volume from Figure 8d,e show a higher standard deviation when 2.7 < *x* < 1.3 [in Na_*x*_V_2_(PO_4_)_3_]. In these regions, the content of the intermediate phase is quite low, which gives rise to less intense and border reflections.

## 4 Conclusions

Here, the structural characteristics of Na_3_V_2_(PO_4_)_3_ coated with carbon are examined using synchrotron-based powder X-ray diffraction. We observe different structural behaviors during charge and discharge. During charging, the system goes through two biphasic transitions, first from Na_3_V_2_(PO_4_)_3_ to Na_2_V_2_(PO_4_)_3_ followed by a transition into NaV_2_(PO_4_)_3_, while discharge only shows a single two-phase transition. Despite the biphasic reaction mechanism and significant volume variations in the unit cell, Na_3_V_2_(PO_4_)_3_ maintains its structural stability during cycling according to the electrochemical and *operando* PXRD data. The ceramic solid electrolyte Na_3.16_Zr_1.84_Y_0.16_Si_2_PO_12_ is prepared by an association of tape-casting and hot pressing at low pressure and low temperature. The NVP/C||Na_3.16_Zr_1.84_Y_0.16_Si_2_PO_12_||Na cell features a solid electrolyte layer that is several micrometers thick and exhibits excellent cycling performance. The solid-state cell shows a remarkable reversible capacity of 95 mAh/g (at C/10), and cycling retention is 78.3% after 1100 cycles at room temperature. In conclusion, this work describes a viable method for formulating excellent solid electrolytes with improved safety, which is beneficial for the industrialization of high-performance all-solid-state batteries in the near future.

## Supporting Information Available

Material characterizations, all scans (scan nos. 8 to 63) with nonscaled and scaled backgrounds to compensate for fluctuations in the beam intensity from the electron top-up method in synchrotron storage rings, EDS spectrum of NVP/C powder, Rietveld refinement of the *ex situ* diffraction pattern of the pristine NVP/C powder mounted in a Kapton capillary, EDS analysis table of NVP/C sample, and comparison table of reported solid-state sodium-ion batteries based on Na_3_V_2_(PO_4_)_3_ electrode (PDF)
