# Supplementary material for: Na3V2(PO4)3 Cathode for Room-Temperature Solid-State Sodium-Ion Batteries: Advanced In Situ Synchrotron X-ray Studies to Understand Intermediate Phase Evolution
Source: Chem Mater. 2024 Feb 20;36(5):2314–24. doi: 10.1021/acs.chemmater.3c02585 (PMC10938495; doi:10.1021/acs.chemmater.3c02585)
Supplement: Supplementary file 1 — cm3c02585_si_001.pdf [file cm3c02585_si_001.pdf]

Supporting Information for:

$Na_3V_2(PO_4)_3$ cathode for Room-Temperature Solid-State Sodium-Ion Batteries: Advanced *In Situ* Synchrotron X-ray Studies to Understand Intermediate Phase Evolution

Bidhan Pandit [a,*], Morten Johansen [b], Cynthia Susana Martínez-Cisneros [a], Johanna M. Naranjo-Balseca [a], Belen Levenfeld [a], Dorthe Bomholdt Ravnsbæk [b], Alejandro Varez [a,*]

[a] *Department of Materials Science and Engineering and Chemical Engineering, Universidad Carlos III de Madrid, Avenida de la Universidad 30, 28911 Leganés, Madrid, Spain*

[b] *Centre for Integrated Materials Research, Department of Chemistry, Aarhus University, Langelandsgade 140, DK-8000, Aarhus, Denmark*

***Corresponding authors**

E-mail address: bpandit@ing.uc3m.es, physics.bidhan@gmail.com (B. Pandit); alvar@ing.uc3m.es (A. Varez)

S1. Material characterizations

Cu-Kα radiation (λ = 1.5406 Å) was used in the Bruker AXS D8 Advance for the XRD investigation with a diffraction angle (2θ) range of 10–80°. XPS (PHI 5000 VersaProbe II ULVAC INC) was used to analyze the oxidation states of the elements associated with the electrode material. In a JEOL Model JSM − 6390LV, the surface morphology and related composition were examined using Field emission scanning electron microscopy (FESEM) and energy dispersive X-ray (EDX) spectroscopy. A LaB6 source and model JEOL 2100 were used for High-resolution transmission electron microscopy (HRTEM) analysis.

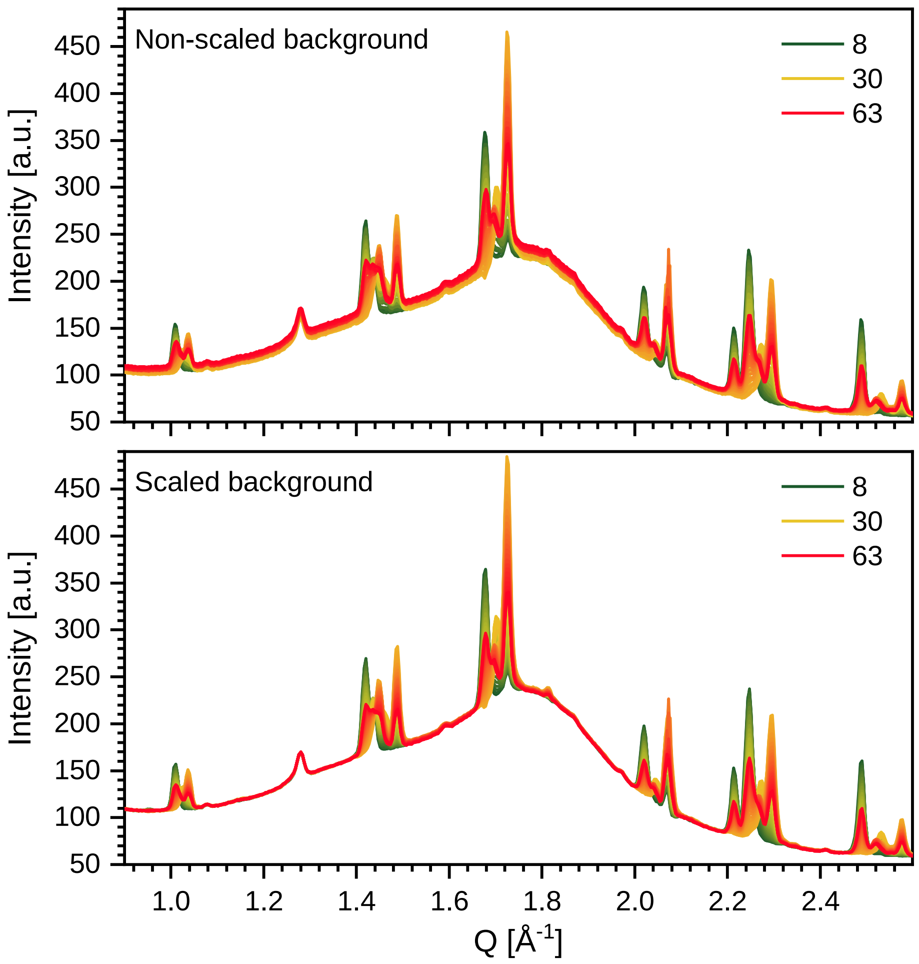

Fig. S2. All scans from 8-63 as background scaled and un-scaled data showing that relative intensities are unchanged in the scaled data however the background in constant throughout scans.

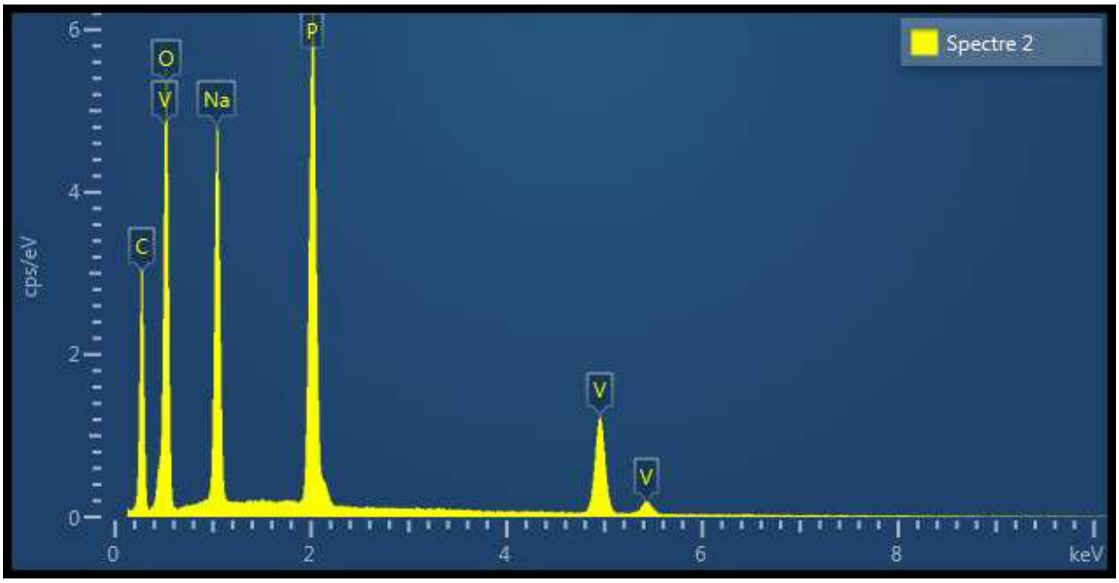

Fig. S3. (a) EDS spectrum of NVP/C.

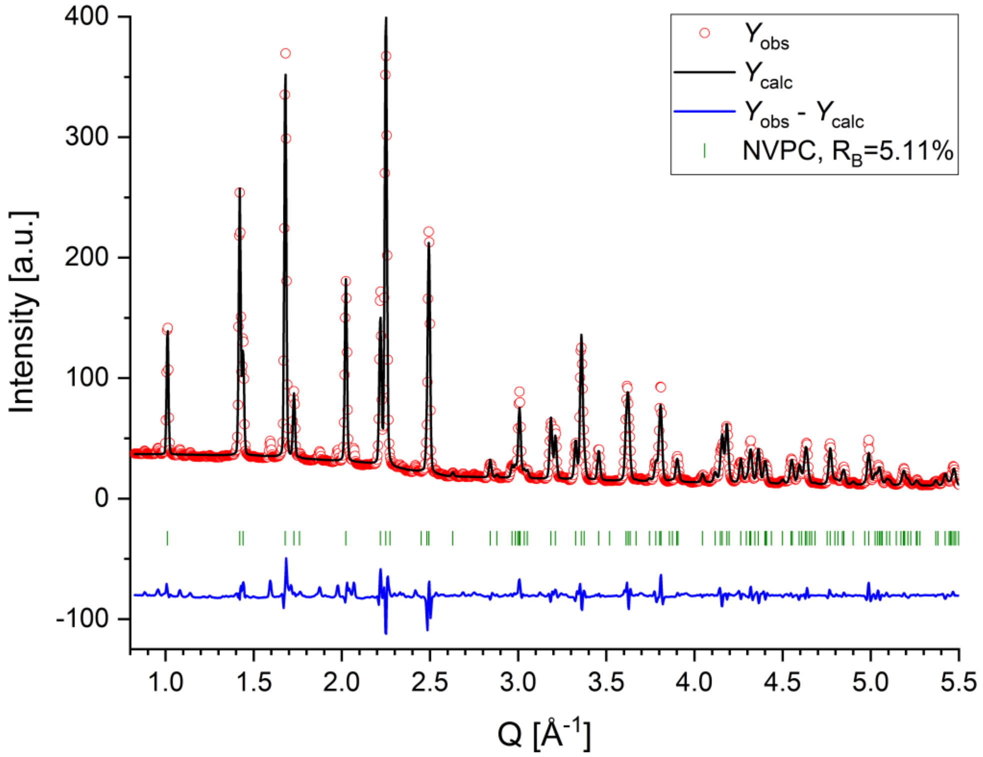

Fig. S4. Rietveld refinement of the *ex situ* diffraction pattern of the pristine $Na_3V_2(PO_4)_3/C$ powder mounted in a Kapton capillary. A particle size of ~140 nm has been calculated based on single peak analysis using the Scherrer equation.

Tables

Table T1. EDS analysis of NVP/C sample

Elements	% Mass	% Atomic
C	20.05	33.98
O	29.25	37.22
Na	9.30	8.23
P	15.60	10.25
V	25.81	10.31
Total	100.00	100.00

Table T2. Electrochemical behaviors of various solid-state sodium ion batteries based on $Na_3V_2(PO_4)_3$ electrode

Battery structure	Operating temperature (°C)	Charge-discharge		Stability		Ref.
		Capacity (mAh/g)	Current rate	Retention (%)	Cycles	
$Na_3V_2(PO_4)_3$|$Na_2Zn_2TeO_6$|Na	80	50	0.2 C	~100	10	[1]
$Na_3V_2(PO_4)_3$|NZTO-$C_{0.02}$|Na	80	21	0.2 C	~33	20	[2]
$Na_3V_2(PO_4)_3$|CPE|Na	70	85	0.5 C	94.1	350	[3]
$Na_3V_2(PO_4)_3$|CPE-ILO|Na	60	30	2 C	86.7	70	[4]
$Na_3V_2(PO_4)_3$/C|$Na_{3.16}Zr_{1.84}Y_{0.16}Si_2PO_{12}$|Na	Room temperature	95	0.1 C	78.3	1100	Present work

References

(1) Li, Y.; Deng, Z.; Peng, J.; Chen, E.; Yu, Y.; Li, X.; Luo, J.; Huang, Y.; Zhu, J.; Fang, C.; Li, Q.; Han, J.; Huang, Y. A P2-Type Layered Superionic Conductor Ga-Doped Na 2 Zn 2 TeO 6 for All-Solid-State Sodium-Ion Batteries. *Chem. - A Eur. J.* **2018**, *24* (5), 1057–1061. https://doi.org/10.1002/chem.201705466.

(2) Deng, Z.; Gu, J.; Li, Y.; Li, S.; Peng, J.; Li, X.; Luo, J.; Huang, Y.; Fang, C.; Li, Q.; Han, J.; Huang, Y.; Zhao, Y. Ca-Doped Na2Zn2TeO6 Layered Sodium Conductor for All-Solid-State Sodium-Ion Batteries. *Electrochim. Acta* **2019**, *298*, 121–126. https://doi.org/10.1016/j.electacta.2018.12.092.

(3) Zhang, X.; Wang, X.; Liu, S.; Tao, Z.; Chen, J. A Novel PMA/PEG-Based Composite Polymer Electrolyte for All-Solid-State Sodium Ion Batteries. *Nano Res.* **2018**, *11* (12), 6244–6251. https://doi.org/10.1007/s12274-018-2144-3.

(4) Chen, G.; Bai, Y.; Gao, Y.; Wang, Z.; Zhang, K.; Ni, Q.; Wu, F.; Xu, H.; Wu, C. Inhibition of Crystallization of Poly(Ethylene Oxide) by Ionic Liquid: Insight into Plasticizing Mechanism and Application for Solid-State Sodium Ion Batteries. *ACS Appl. Mater. Interfaces* **2019**, *11* (46), 43252–43260. https://doi.org/10.1021/acsami.9b16294.
